# Interplay between genome-wide implicated genetic variants and environmental factors related to childhood antisocial behavior in the UK ALSPAC cohort

**DOI:** 10.1007/s00406-018-0964-5

**Published:** 2018-12-19

**Authors:** I. Hyun Ruisch, Andrea Dietrich, Jeffrey C. Glennon, Jan K. Buitelaar, Pieter J. Hoekstra

**Affiliations:** 1 Department of Child and Adolescent Psychiatry, University of Groningen, University Medical Center Groningen, Hanzeplein 1, 9713GZ Groningen, The Netherlands; 2 Department of Cognitive Neuroscience, Donders Institute for Brain, Cognition and Behaviour, Radboud University Medical Center, Geert Grooteplein Zuid 10, 6525GA Nijmegen, The Netherlands; 3 Karakter Child and Adolescent Psychiatry University Centre, Reinier Postlaan 12, 6525GC Nijmegen, The Netherlands

**Keywords:** ALSPAC, Antisocial behavior, Aggression, Gene–environment interaction, Maltreatment, Smoking during pregnancy

## Abstract

We investigated gene–environment (G × E) interactions related to childhood antisocial behavior between polymorphisms implicated by recent genome-wide association studies (GWASs) and two key environmental adversities (maltreatment and smoking during pregnancy) in a large population cohort (ALSPAC). We also studied the *MAOA* candidate gene and addressed comorbid attention-deficit/hyperactivity disorder (ADHD). ALSPAC is a large, prospective, ethnically homogeneous British cohort. Our outcome consisted of mother-rated conduct disorder symptom scores at age 7;9 years. G × E interactions were tested in a sex-stratified way (*α* = 0.0031) for four GWAS-implicated variants (for males, rs4714329 and rs9471290; for females, rs2764450 and rs11215217), and a length polymorphism near the *MAOA-*promoter region. We found that males with rs4714329-GG (*P* = 0.0015) and rs9471290-AA (*P* = 0.0001) genotypes were significantly more susceptible to effects of smoking during pregnancy in relation to childhood antisocial behavior. Females with the rs11215217-TC genotype (*P* = 0.0018) were significantly less susceptible to effects of maltreatment, whereas females with the *MAOA*-HL genotype (*P* = 0.0002) were more susceptible to maltreatment effects related to antisocial behavior. After adjustment for comorbid ADHD symptomatology, aforementioned G × E’s remained significant, except for rs11215217 × maltreatment, which retained only nominal significance. Genetic variants implicated by recent GWASs of antisocial behavior moderated associations of smoking during pregnancy and maltreatment with childhood antisocial behavior in the general population. While we also found a G × E interaction between the candidate gene *MAOA* and maltreatment, we were mostly unable to replicate the previous results regarding *MAOA*–G × E’s. Future studies should, in addition to genome-wide implicated variants, consider polygenic and/or multimarker analyses and take into account potential sex stratification.

**Electronic supplementary material:**

The online version of this article (10.1007/s00406-018-0964-5) contains supplementary material, which is available to authorized users.

## Introduction

Conduct disorder (CD) is a pediatric behavioral disorder with a prevalence of approximately 4–16% in boys and about 1–9% in girls [1]. Hallmark of CD are antisocial behaviors, that is, serious violations of basic rights of other people and/or age-appropriate societal norms resulting in severe aggression, deceitfulness, and rule-breaking behavior. The severe nature of these symptoms gives rise to a significant burden for affected patients, families, and societies at large [2]. About 40% of boys and 25% of girls with CD are estimated to display antisocial behaviors persisting into adulthood and develop antisocial personality disorder [3, 4].

CD symptomatology or antisocial behaviors can be considered as continuous traits that are caused by both genetic and environmental risk factors. More specifically, the interplay between genes and environment, also known as gene–environment (G × E) interactions, can provide insight into why some individuals are more susceptible to certain adverse genetic or environmental factors than others. These G × E interactions are assumed to be of great importance in multifactorial traits such as antisocial behavior [5–7].

Recent insights suggest that the classic candidate G × E literature holds important limitations. Most notable, the use of poorly replicated candidate genes, underpowered samples, and inappropriate correction for multiple comparisons are suspected to have resulted in an inflated rate of false-positive findings across studies [8–10]. Instead, hypothesis-free, genome-wide association studies (GWASs) can overcome these issues and thereby provide more robust candidates for both gene discovery and G × E research [8, 11]. Regarding GWAS literature, two large studies identified a number of novel, sex-stratified susceptibility loci for antisocial behavior and antisocial personality disorder recently [12, 13]. Moreover, a G × E interaction was suggested between one of these loci and childhood familial difficulties in males within the general population [13].

So far, one of the most studied G × E interactions in relation to antisocial behavior involves a 30 bp length polymorphic region (LPR) in the *monoamine oxidase A (MAOA)* gene and exposure to childhood maltreatment. The LPR affects the functionality of the MAOA enzyme resulting in alleles with lower (*MAOA-L*) and higher (*MAOA-H*) activity [5, 6, 14]. Since the *MAOA* gene is located on the *X* chromosome, males have only one copy of the gene, whereas females have two, supporting sex-stratified analyses. Indeed, meta-analytic evidence has suggested that males with the *MAOA-L* genotype were more susceptible to effects of maltreatment than males with *MAOA-H*, while females with the *MAOA-H* genotype appeared to be more susceptible to maltreatment effects, albeit weakly and less consistent than in males [15]. Similar sex-stratified G × E interaction patterns in relation to antisocial behavior have been reported for *MAOA* × maternal smoking during pregnancy (i.e., males with *MAOA-L* were more susceptible to effects of smoking than males with *MAOA-H*, whereas females with *MAOA-H* were more susceptible to effects of smoking than females with *MAOA-L*) [16]. Thus, given location on the *X* chromosome and (meta-analytic) implication of different functional alleles, more sex-stratified research is needed, taking into account limitations in candidate G × E research.

Regarding sex differences related to antisocial behavior, higher rates of antisocial behavior and crime have been reported in males compared to females [17]. Furthermore, males appear to be over-represented in clinical samples [1]. Considering these observations, sex-stratified investigation of potential risk factors is very much needed. Further reasons for conducting sex-stratified analyses include recent GWAS results pointing to different susceptibility loci for antisocial behavior in males and females [12, 13].

Moreover, another important yet frequently overlooked limitation of a substantial part of the G × E literature arises from a lack of covariate interaction modelling in the G × E analyses [18]. Modelling covariate interactions is important, because both the genetic and environmental factor of interest might be moderated by control variables and the G × E interaction should be adjusted accordingly. Another point of consideration is the inconsistent control for the highly comorbid attention-deficit/hyperactivity disorder (ADHD), which may actually drive part of the associations reported with antisocial behavior [1, 19]. Similarly, further improvements could also be made by adjusting for frequently comorbid internalizing problems [1]. Furthermore, gene–environment correlations (i.e., genetic confounding of the environment) should also be taken into account as a potential driving force behind apparent G × E interactions [20].

In this study, we aimed to address the aforementioned issues concerning the existing G × E literature and investigated G × E interactions in relation to childhood antisocial behavior in the well-powered Avon Longitudinal Study of Parents and Children (ALSPAC). We focused on two key environmental risk factors for antisocial behavior, namely maternal smoking during pregnancy and childhood maltreatment [7, 15, 16, 21], in the interplay with recently identified genetic variants from GWASs of antisocial behavior, while controlling for potential confounding by comorbid ADHD and addressing specific statistical concerns. Furthermore, we aimed to replicate previously reported G × Es for the much studied *MAOA* candidate gene.

## Methods

### The ALSPAC sample

ALSPAC is an ongoing, prospective, longitudinal birth cohort, which initially recruited 14,541 pregnant women in Avon, UK with expected delivery dates from April 1991 to December 1992 and their subsequently born children. At the time of recruitment, mothers were between age 16 and 45 and represented about 85% of pregnant women in the catchment area. When children reached the age of 7, the initial sample was enriched with eligible cases who had failed to join the study initially. This resulted in an enrollment of 713 additional children. Longitudinally collected data comprise a wide range of phenotypic and environmental measures, as well as biological samples and (epi)genetic data. Further details regarding recruitment, study design, and generalizability have been reported elsewhere [22–24]. Ethical approval for the ALSPAC study was obtained from the ALSPAC Ethics and Law Committee as well as the Local Research Ethics Committees. Details on the ethics committee’s and institutional review boards that approved the study can be found at http://www.bristol.ac.uk/alspac/researchers/research-ethics/. For the present study, we included subjects with data on smoking during pregnancy, maltreatment, childhood antisocial behavior, and genotype data for a specific set of candidate polymorphisms. We only included subjects with a Caucasian ethnicity.

### Main outcome: childhood antisocial behavior

As our main outcome, we used childhood antisocial behavior as measured by mother-rated CD symptom scores. Assessments were carried out using the Development and Well-Being Assessment (DAWBA) [25] at the age of 7 years and 9 months. The DAWBA is a psychiatric-diagnostic interview with the parents assessing psychopathology in children and adolescents with good validity [25]. Individual symptoms, derived from the Diagnostic and Statistical Manual of Mental Disorders (DSM) version IV [26], were rated on a three-point scale (0–2). Possible CD symptom scores ranged from 0 to 14. To reduce excess variance and avoid low cell counts given the skewed score distribution, we recoded 32 males with a score > 4 as 4, and 35 females with a score > 3 as 3. Supplementary Table S3 provides frequency tables of childhood antisocial behavior scores.

### Genotypes

Details regarding genotyping quality control procedures in the ALSPAC sample are described elsewhere [27]. In summary, genotyping of single-nucleotide polymorphisms (SNPs) was carried out by the Wellcome Trust Sanger Institute, Cambridge, UK, and the Laboratory Corporation of America, Burlington, NC, US, using the Illumina HumanHap550 beadchip array and subsequent quality control filters regarding SNP call rate (0.95), subject call rate (0.97), evidence of Hardy–Weinberg violation (*P* cut-off 5.00E − 07), minor allele frequency (0.01), and autosomal heterozygosity (outliers were removed). Furthermore, imputation was performed with Impute2 v2.2.2 software, using the 1000 Genomes phase 1 (version 3) reference panel, and subsequent filtering based on Impute Information scores (only SNPs with Info > 0.8 were retained). Our data set obtained from the ALSPAC study included genotype data for 8941 children. Using the PLINK software [28, 29], we extracted four top SNPs from two GWASs. That is, from study one [13], we included rs4714329 (chromosome 6:40273457, G/A, G effect allele) and rs9471290 (chromosome 6:40260515, A/G, A effect allele), which appeared to be mainly a male-driven signal. From study two [12], we included rs2764450 (chromosome 1:180242092, T/C, T effect allele) and rs11215217 (chromosome 11:114689701, T/C, T effect allele), which were identified in female-only analyses. Given, sex-stratified implication, we performed sex-stratified G × E analyses in our sample (i.e., investigating rs4714329 and rs9471290 in males, and rs2764450 and rs11215217 in females). Supplementary Table S1 lists genotype statistics for the included SNPs. Since rs4714329 and rs9471290 were both located within the chromosome 6p21.2 region, we used the web-based application suite LD link (available at https://analysistools.nci.nih.gov/LDlink/) to obtain an estimate of linkage disequilibrium in the European populations. Furthermore, the male-only SNP G × E’s were initially contrasted as an additive model (0/1/2 effect alleles coded as 0/1/2). However, since the data indicated a recessive model (0/1/2 effect alleles coded as 0/0/1) as a better fit, this model was used to contrast the genotypes for the male-only SNPs instead. The extra tests conducted were addressed in the multiple testing corrections applied (see Sect. “Statistical analyses”). Results for male-only SNP G × Es tested as an additive model are provided in Supplementary Table S2. For the female-only SNPs, the cell counts for the T-allele homozygotes were very small (please see Supplementary Table S1 for genotype statistics); therefore, we contrasted these SNPs as a heterozygote model without the T-allele homozygotes.

In addition to GWAS-implicated SNPs, we also investigated a 30 bp length polymorphic region (LPR) near the promoter region of the *monoamine oxidase A (MAOA)* gene. Therefore, in addition to SNP data, we investigated LPR-genotype data for *MAOA*, which was available for 9467 subjects. Variants with 2, 3, and 5 repeats were coded as low-activity alleles (*MAOA-L*), whereas variants with 3.5 and 4 repeats were coded as high-activity alleles (*MAOA-H*) [30, 31]. As mentioned, *MAOA* is located on the *X* chromosome, and therefore, males are hemizygous, while females have two gene copies. As the *MAOA-*LPR has been implicated in both males and females [15], we investigated its effect in both sexes. Because the extent of *X* inactivation at the *MAOA* locus is, however, unclear [32, 33], there remains some debate whether or not to include females with both low- and high-activity alleles [15]. Therefore, we contrasted the *MAOA-*LPR for males as a hemizygous model for the low-activity allele (*MAOA-L*) and for females as an additive model for the number of high-activity alleles (*MAOA-H*) [15].

To investigate population stratification, we merged our genome-wide SNP data with the 1000 Genomes phase 1 reference data set [34], which contains data from 14 different global populations. We then analyzed whether the genetic principal components showed the evidence of population structure by mapping our subjects onto the known populations of the 1000 Genomes data set. In addition, any subjects in our data set scoring less than − 2 or more than + 2 standard deviations on any of the first ten principal components (using only European reference populations from the 1000 Genomes data set) were excluded from the analyses.

### Environmental adversities

Maternal smoking during pregnancy was assessed by maternal self-report questionnaires at 18 weeks gestation and was defined as any versus no maternal tobacco smoking during pregnancy. This included the use of cigarettes, cigars, pipes, and other forms of tobacco smoking. Childhood maltreatment consisted of mother reported assessments at multiple time points between birth and 7;9 years of physical (available at 1;6, 1;9, 2;6, 2;9, 3;6, 4;9, 5;1, 5;9, 6;1, 6;9 years), sexual (available at 1;6, 2;6, 3;6, 4;9, 5;9, 6;9 years), or emotional abuse (available at 0;8, 1;9, 2;9, 5;1, 6;1 years) and maladaptive parenting (available at 1;6, 1;9, 2;6, 2;9, 3;6, 3;11, 4;9, 5;9, 6;1, 6;6, 6;9, 7;1 years), the last of which was defined as hitting of, shouting at, or a hostile attitude towards the child. If, at any time point, any type of abuse occurred and affected the child ‘much’ or ‘moderate’, abuse was coded as being present. Hitting and shouting were coded as present at a given time point when they were reported as occurring ‘often’ or ‘sometimes’. Hostility was coded as present at a given time point when the mother responded positively to ‘being often irritated by the child’, ‘having battles of will with the child’ or ‘the child gets on the nerves of mother’. If both at preschool (any time point between 0 and 5 years) and school age (any time point between 5 and 7;9 years), hitting, shouting, or hostility was reported; maladaptive parenting was coded as being present. When abuse, maladaptive parenting, or both were present, maltreatment was coded as present. Otherwise, maltreatment was coded as absent. Definition of maltreatment was considered broadly across multiple time points to obtain a global measure covering the childhood period up to 7;9 years, similar to the construct used by the study of Lereya et al. [35]. Please note that the ALSPAC website contains the details of all the data that are available through a fully searchable data dictionary and variable search tool (http://www.bristol.ac.uk/alspac/researchers/our-data).

### Statistical analyses

Calculation of genotype statistics and principal component analyses was done using the PLINK software [28, 29]. Our main analyses were modelled in R [36], using the regression implementation from the ‘MASS’ package [37]. Given the positively skewed and over dispersed outcome data, we used negative binomial regression [38, 39]. Negative binomial regression uses a log-link function, and when regression coefficients are exponentiated, an incidence rate ratio (IRR) is obtained. The IRR gives the ratio of antisocial behavior scores between subjects with the predictor (i.e., risk genotype and risk environment) present versus absent. For example, an IRR of 1.50 indicates that at-risk subjects are predicted to have a 50% higher antisocial behavior score than other subjects. To control the family-wise error rate for multiple comparisons, we divided the nominal significance threshold by the number of tests carried out (for males, we carried out 10 tests, and for females, we carried out 6 tests, totaling 16 tests), resulting in a corrected alpha of 0.05/16 ≈ 0.0031.

Given that our outcome data were measured at the same age for all subjects and we considered sex-stratified G × E models, there was no need to include age or sex as control variables. As preliminary analyses indicated an association with childhood antisocial behavior in our sample, we included the following control variables: presence of a low socioeconomic status (which was determined as the lowest two social classes based on the SOC2000 classification [40]), maternal single-parent status, comorbid ADHD symptom scores (assessment using the DAWBA, possible score ranges 0–36, rated by the mother at age 7;9 years), and comorbid emotional problems (assessment using the Strengths and Difficulties Questionnaire [41], emotional problems subscale, possible score ranges 0–10, rated by mother at age 6;9 years). In addition, we included the first ten genetic principal components. In addition to control variable main effects, control variable interaction terms with both the genetic and environmental factor were included in each G × E model to more robustly control for confounding effects [18]. Furthermore, as G × E interactions may be confounded by gene–environment correlations, we also investigated correlations between our genetic variants and environmental factors. Sensitivity analyses were conducted to investigate potential confounding effects by comorbid ADHD symptoms and comorbid emotional problems.

## Results

### Descriptive statistics

**Fig. 1:**
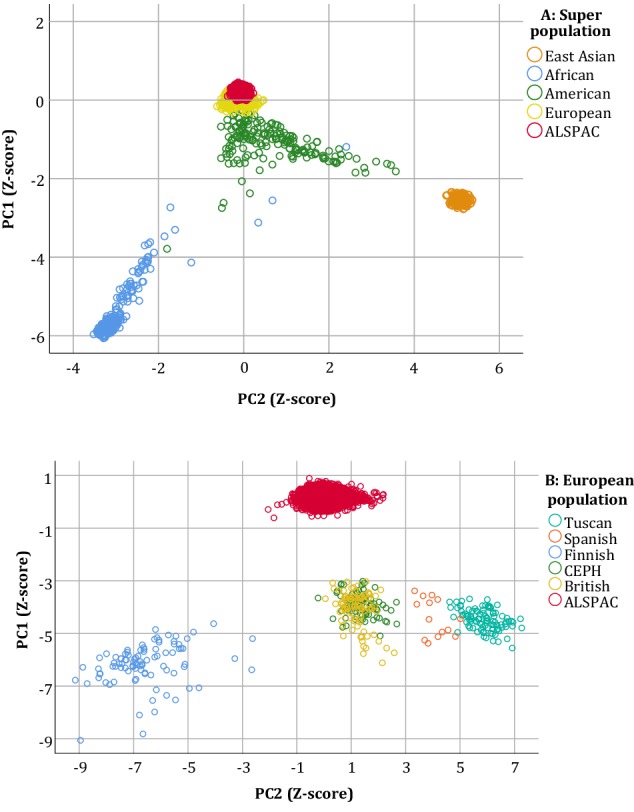
Genetic principal component analysis. Scatterplots of the first two genetic principal components of ALSPAC merged with the 1000 Genomes phase 1 data set. **a** Clustering of superpopulations from different continents, whereas **b** shows clustering of populations within Europe. *PC1* principal component (1), *PC2* principal component (2), *SD* standard deviation, *CEPH* Centre d’Etude du Polymorphisme Humain

Table 1 provides sex-stratified descriptive and summary statistics for our sample. Supplementary Table S1 provides the minor allele frequency, Hardy–Weinberg Equilibrium test, call rate, and all genotype frequencies for the included SNPs. Linkage disequilibrium was estimated to be moderate between the two SNPs on chromosome 6 (rs4714329 and rs9471290; *D*′ = 0.77 and *r*^2^ = 0.451). Results from the principal component analysis showed that the ALSPAC subjects constituted a homogeneous sample, both in relation to global and European populations (Fig. 1a, b). Within Europe, ALSPAC appeared to be most proximal to the British and Centre d’Etude du Polymorphisme Humain (CEPH) subclusters of individuals from the 1000 Genomes data set (Fig. 1b).

**Table 1:** Descriptive and summary statistics

	Males (*N* = 2547 max.) *N* (%) or mean ± SD	Females (*N* = 2395 max.) *N* (%) or mean ± SD
Age at outcome 7;9 years	2547 (100%)	2395 (100%)
Caucasian ethnicity (self-report)	2547 (100%)	2395 (100%)
Childhood antisocial behavior score^#^	0.60 ± 1.10 (range 0–10)	0.46 ± 0.87 (range 0–8)
Childhood ADHD score^#^	5.83 ± 7.37 (range 0–36)	3.45 ± 5.32 (range 0–35)
Childhood emotional problems score	1.41 ± 1.64 (range 0–9)	1.53 ± 1.66 (range 0–10)
Smoking during pregnancy	428 (16.80%)	394 (16.46%)
Maltreatment^#^	892 (62.33%)	712 (54.85%)
MAOA-L (males)/HL (females)	765 (34.23%)	969 (46.36%)
MAOA-HH (females)		858 (41.05%)
rs4714329 GG	398 (15.63%)	–
rs9471290 AA	321 (12.60%)	–
rs2764450 TC	–	280 (11.75%)
rs11215217 TC	–	292 (12.32%)
Low socioeconomic status	417 (16.37%)	375 (15.66%)
Single-parent status	81 (3.18%)	88 (3.67%)

*MAOA* monoamine oxidase A, *MAOA-L*/*H MAOA* low-/high-activity allele, *ADHD* attention-deficit/hyperactivity disorder

^#^Significant difference between males and females, *α* = 0.0071 for comparing seven variables (antisocial score, ADHD score, emotional problems score, smoking during pregnancy, maltreatment, low socioeconomic status, and single-parent status) between males and females

### Gene–environment correlations

No gene–environment correlations were observed between any of the polymorphisms and the environmental factors (see Supplementary Table S4 for results).

### G × E interactions in relation to childhood antisocial behavior in males

**Fig. 2:**
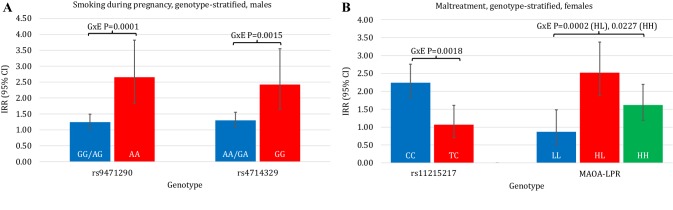
Sex- and genotype-stratified effects of environmental adversities in relation to childhood antisocial behavior. Genotype-moderated effects of smoking during pregnancy in males (**a**) and maltreatment in females (**b**). *IRR* incidence rate ratio, *MAOA* monoamine oxidase A, *LPR* length polymorphic region, *MAOA-L/H MAOA* low-/high-activity allele, Error bars show 95% confidence intervals for the IRR. Gene–environment (G × E) interaction *P* values are shown on top

See Table 2 and Fig. 2 for all results (the final number of included subjects for each analysis is also provided in Table 2). Main effects were observed for both smoking during pregnancy [*P* < 0.0001 (*N* = 2547)] and childhood maltreatment [*P* < 0.0001 (*N* = 1431)], yet none of the genetic polymorphisms showed a main effect. For rs4714329 and rs9471290, effects of smoking during pregnancy were strongest in G- and A-allele homozygotes [significant positive G × E interactions *P* = 0.0015 (N = 2547) and 0.0001 (N = 2547), respectively]. No interactions between these SNPs and maltreatment were found. For *MAOA-*LPR, no G × E interactions were seen with smoking during pregnancy or maltreatment. Table S5 provides sex- and genotype-stratified environmental main effects in relation to childhood antisocial behavior.

**Table 2:** G × E interactions in relation to childhood antisocial behavior in males and females

Contrast	Males			Females		
	*N*	IRR (95% CI)	*P*	*N*	IRR (95% CI)	*P*
Smoking during pregnancy	2547	1.43 (1.22–1.68)	< 0.0001*	2394	1.78 (1.51–2.09)	< 0.0001*
Maltreatment	1431	1.97 (1.65–2.35)	< 0.0001*	1298	1.99 (1.66–2.40)	< 0.0001*
MAOA-L (males)/HL (females)	2235	1.01 (0.87–1.17)	0.91	2090	1.03 (0.81–1.31)	0.80
MAOA-HH (females)					1.08 (0.85–1.37)	0.54
rs4714329 GG	2547	0.95 (0.79–1.15)	0.63			
rs9471290 AA	2547	1.16 (0.96–1.41)	0.13			
rs2764450 TC				2383	1.07 (0.86–1.33)	0.56
rs11215217 TC				2370	0.97 (0.80–1.18)	0.76
MAOA-L (males)/HL (females) × smoking during pregnancy	2235	1.00 (0.70–1.43)	0.99	2089	1.15 (0.63–2.10)	0.64
MAOA-HH (females) × smoking during pregnancy					1.23 (0.68–2.25)	0.49
MAOA-L (males)/HL (females) × maltreatment	1266	1.35 (0.90–2.03)	0.15	1135	3.27 (1.74–6.14)	0.0002*
MAOA-HH (females) × maltreatment					2.09 (1.11–3.93)	**0.0227**
rs4714329 GG × smoking during pregnancy	2547	1.95 (1.29–2.94)	0.0015*			
rs4714329 GG × maltreatment	1431	0.76 (0.47–1.25)	0.28			
rs9471290 AA × smoking during pregnancy	2547	2.18 (1.47–3.24)	0.0001*			
rs9471290 AA × maltreatment	1431	1.28 (0.71–2.31)	0.42			
rs2764450 TC × smoking during pregnancy				2382	1.22 (0.75–1.97)	0.43
rs2764450 TC × maltreatment				1291	1.61 (0.89–2.91)	0.11
rs11215217 TC × smoking during pregnancy				2369	0.69 (0.40–1.19)	0.18
rs11215217 TC × maltreatment				1285	0.47 (0.29–0.75)	0.0018*

All analyses were adjusted for socioeconomic status, single-parent status, and the first ten genetic principal components, including covariate interaction terms for the G × E models

Significance values are in bold (*P* < 0.05)

*G × E* gene-by-environment interaction, *IRR* incidence rate ratio, *MAOA* monoamine oxidase A, *MAOA-L*/*H MAOA* low-/high-activity allele

*Significant (i.e., corrected for multiple hypotheses) at *α* = 0.0031

### G × E interactions in relation to childhood antisocial behavior in females

See Table 2 and Fig. 2 for all results (the final number of included subjects for each analysis is also provided in Table 2). Main effects were observed for both smoking during pregnancy [*P* < 0.0001 (N = 2394)] and childhood maltreatment [*P* < 0.0001 (N = 1298)], yet none of the genetic polymorphisms showed a main effect. For rs11215217, we found that the effects of maltreatment were less strong in TC heterozygotes [a significant negative G × E interaction *P* = 0.0018 (N = 1285)]. No interaction with smoking during pregnancy was observed. For rs2764450, no G × E interactions with smoking during pregnancy or maltreatment were observed. Regarding *MAOA-*LPR, no G × E interaction was seen with smoking during pregnancy, whereas subjects with both high- and low-activity alleles appeared to be most susceptible to effects of maltreatment [a significant positive G × E interaction, *P* = 0.0002 (N = 1135)]. Table S5 provides sex- and genotype-stratified environmental main effects in relation to childhood antisocial behavior.

### Sensitivity analyses addressing potential confounding by comorbid ADHD symptoms

See Table 3 for all results (the final number of included subjects for each analysis is also provided in Table 3). After adjusting significant G × E interactions for comorbid ADHD symptom scores, we found that the G × Es of rs4714329-GG and rs9471290-AA with smoking during pregnancy (*P* = 0.0014 and *P* = 0.0010, respectively, males), as well as the G × E between *MAOA-*LPR and maltreatment remained significant (*P* = 0.0013, females). The G × E between rs11215217-TC and maltreatment only reached nominal significance after adjustment for comorbid ADHD symptoms (*P* = 0.0074, females).

**Table 3:** Sensitivity analyses: adjustment for comorbid ADHD symptomatology

Contrast	*N*	IRR (95% CI)	*P*
MAOA-HL (females) × maltreatment	1129	2.74 (1.48–5.08)	0.0013*
MAOA-HH (females) × maltreatment		1.98 (1.07–3.66)	0.0298
rs4714329 GG (males) × smoking during pregnancy	2529	1.85 (1.27–2.70)	0.0014*
rs9471290 AA (males) × smoking during pregnancy	2529	1.87 (1.29–2.71)	0.0010*
rs11215217 TC (females) × maltreatment	1278	0.53 (0.33–0.84)	0.0074

All analyses were adjusted for socioeconomic status, single-parent status, comorbid ADHD symptom scores, and the first ten genetic principal components, including covariate interaction terms for the G × E models. Comorbid ADHD symptom scores were dichotomized closest to the 67th percentile to reduce collinearity with the covariate interaction terms

*G × E* gene-by-environment interaction, *IRR* incidence rate ratio, *MAOA* monoamine oxidase A, *MAOA-L*/*H MAOA* low-/high-activity allele, *ADHD* attention-deficit/hyperactivity disorder

*Significant (i.e., corrected for multiple hypotheses) at *α* = 0.0031

### Sensitivity analyses addressing potential confounding by comorbid emotional problems

See Table 4 for all results (the final number of included subjects for each analysis is also provided in Table 4). After adjusting significant G × E interactions for comorbid emotional problems scores, we found that the G × E’s of rs4714329-GG and rs9471290-AA with smoking during pregnancy (*P* = 0.0021 and *P* = 0.0013, respectively, males), as well as the G × E’s of *MAOA-*LPR and rs11215217-TC with maltreatment remained significant (*P* = 0.0006 and *P* = 0.0020, respectively, females).

**Table 4:** Sensitivity analyses: adjustment for comorbid emotional problems

Contrast	*N*	IRR (95% CI)	*P*
MAOA-HL (females) × maltreatment	1095	2.97 (1.59–5.56)	0.0006*
MAOA-HH (females) × maltreatment		1.94 (1.02–3.67)	0.0427
rs4714329 GG (males) × smoking during pregnancy	2358	1.92 (1.27–2.91)	0.0021*
rs9471290 AA (males) × smoking during pregnancy	2358	1.97 (1.30–2.96)	0.0013*
rs11215217 TC (females) × maltreatment	1239	0.45 (0.27–0.74)	0.0020*

All analyses were adjusted for socioeconomic status, single-parent status, comorbid emotional problems scores, and the first ten genetic principal components, including covariate interaction terms for the G × E models. Comorbid emotional problem scores were dichotomized closest to the 67th percentile to reduce collinearity with the covariate interaction terms

*G × E* gene-by-environment interaction, *IRR* incidence rate ratio, *MAOA* monoamine oxidase A, *MAOA-L*/*H MAOA* low-/high-activity allele, *ADHD* attention-deficit/hyperactivity disorder

*Significant (i.e., corrected for multiple hypotheses) at *α* = 0.0031

## Discussion

In this study, we performed sex-stratified analyses of G × E interactions in relation to childhood antisocial behavior in a large population cohort for recent GWAS-implicated SNPs and *MAOA* with two well-known environmental risk factors, namely maternal smoking during pregnancy and childhood maltreatment. Regarding males, our most important findings are that G-allele homozygotes of the rs4714329 SNP and A-allele homozygotes of the rs9471290 SNP appeared to be more susceptible to effects of smoking during pregnancy in relation to antisocial behavior. Regarding females, we found that heterozygotes of the rs11215217 SNP appeared to be less susceptible, and carriers of both low- and high-activity allele of the *MAOA-*LPR appeared to be more susceptible to effects of childhood maltreatment in relation to antisocial behavior.

In males, the related SNPs rs4714329 and rs9471290 appeared to moderate the relation between smoking during pregnancy and antisocial behavior in such a way that risk allele homozygotes appeared to be more vulnerable to effects of maternal smoking than the other genotypes. More specifically, in risk allele homozygotes, antisocial behavior scores were more than twice as high in smoking-exposed subjects compared to unexposed subjects. By using the open-access GTEx database (available at https://www.gtexportal.org/home/), the SNP rs4714329 was linked to the expression of nearby genes *LINC00951* and *LRFN2* in the brain [13]. *LRFN2* encodes a protein suggested to be involved in neural developmental processes such as neurite outgrowth and synaptic plasticity [42]. LRFN2 is part of a larger protein class characterized by a leucine-rich repeat domain. Many leucine-rich repeats containing transmembrane proteins are thought to be involved in nervous system development and neurodevelopmental disorders [43, 44]. LRFN2 regulates the post-synaptic PSD-95 complex, and has also been implicated in erythropoiesis, working memory, and autistic features [42, 45–48]. *LINC00951* is an intergenic, long non-protein coding RNA gene, which is also expressed in the brain [13]. While many of these RNAs remain to be characterized, in general, they are assumed to be involved in gene expression regulation at both epigenetic and (post) transcriptional levels as well as other processes such as genomic imprinting [49]. In addition, these RNAs may play an important role in neurodevelopmental disorders [50].

Smoking during pregnancy has been one of the more strongly associated prenatal risk factors in relation to CD [7, 51], although this may, in part, be due to genetic and/or familial confounding [20, 52, 53]. Tobacco smoke consists of a mixture of many chemicals including nicotine, carbon monoxide, polycyclic aromatic hydrocarbons, and heavy metals, all of which may affect the developing fetus by various mechanisms [54–57]. A number of recent studies investigating gene expression patterns in relation to smoking reported the gene *LRRN3* among their top hits of smoking-related differentially expressed genes [58–60]. Similar to *LRFN2, LRRN3* is a leucine-rich repeat domain containing transmembrane protein expressed in the brain, and suggested to play a role in the development and maintenance of the nervous system [61]. Functionally, LRRN3 has been implicated in autism, antidepressant action, and cortical thickness (alterations of which are associated with conduct and psychopathic features) [62–64]. Although the before mentioned studies of smoking did not specifically target effects of smoking during pregnancy and gene expression alterations might be reversible, the reported results suggest that smoking might exert effects on pathways that are also affected by genetic risk factors related to antisocial behavior. Conversely, G × E interplay might be expected, i.e., moderation effects among genotype and environment such as observed in the present study.

Furthermore, as mentioned before, the use of smoking during pregnancy as an exclusively and independent environmental factor has been a point of discussion. As confounding by both genetic and socio-environmental factors has been suggested [20, 52, 53], this could indicate that the observed G × E with smoking during pregnancy may at least in part be a proxy for a gene–gene interaction and/or G × E interaction with the other environmental factors. However, as we did not observe gene–environment correlations between the selected genetic variants and smoking during pregnancy and controlled our analyses for covariate interactions, we at least addressed part of these confounding issues.

Therefore, although the exact nature of the identified G × E interaction with smoking during pregnancy is not clear, both the genetic and environmental factors in this G × E may affect brain development through effects on leucine-rich repeat protein interaction networks thought to be involved in functions such as synapse and neural circuit formation, and thereby predispose offspring for antisocial behavior [43, 44]. This also implies that future studies should also take into account related neural leucine-rich repeat protein (regulatory) genes when attempting to replicate or extent present findings.

A G × E interaction between the SNP rs11215217 and childhood maltreatment was observed in relation to offspring antisocial behavior in females. The nearest gene to this SNP is a non-coding, uncharacterized RNA gene (*LOC105369506*). As before mentioned, multiple (regulatory) functions of non-coding RNA genes have been described and their role in neurodevelopmental disorders highlighted [49, 50]. Of note, when adjusted for comorbid ADHD symptoms, the interaction became only nominally significant, which might indicate that the effect could be partially driven by comorbid ADHD.

Furthermore, the GWAS in relation to antisocial personality disorder by Rautiainen et al. [13] suggested a male-specific interaction between the SNP rs4714329 and childhood familial difficulties (severe conflicts and/or economic difficulties) in the general population [13]. Since we did not find any (male) G × E interactions between maltreatment and rs4714329 (or the related SNP rs9471290), we conclude that this suggested interaction does not appear to extend to childhood maltreatment in relation to pediatric antisocial behavior.

In addition, while interactions between the near-promoter LPR in *MAOA* and childhood maltreatment in relation to antisocial behavior have been reported for both sexes previously [15], we only observed a G × E interaction in females. More specifically, we observed a disadvantage mostly for maltreatment-exposed females with both low- and high-activity alleles (showing antisocial behavior scores more than twice as high compared to unexposed females), which is slightly different from the (additive) H-allele effect suggested in a previous meta-analysis [15]. Furthermore, in males with a low-activity allele, we did not observe any interaction with maltreatment. While this null finding does not replicate previous meta-analytic results [15], the largest study in the aforementioned meta-analysis also failed to find any interaction between *MAOA* and stressful life events in relation to conduct problems, both in males and females [65]. This study was also conducted within ALSPAC; however, important differences with the current study include the use of childhood life event scores instead of a specific measure of maltreatment, and the use of more general behavioral questionnaire data rather than diagnostic assessments of antisocial behavior. In addition to emphasizing our null finding in males, these differences may also explain the different female G × E results compared to the current study. Regarding smoking during pregnancy, we also failed to replicate the previous G × E findings for *MAOA* [16] in both sexes. Therefore, to conclude, while we reported a G × E between *MAOA*-HL and maltreatment in females, we consider our other negative results regarding *MAOA* as a sign to be slightly cautious when interpreting the earlier candidate gene-based G × E studies in this area [8, 10, 18].

### Strengths and limitations

Strengths of the current study have been the use of well-powered GWAS-implicated variants as novel targets for G × E research, the use of a large, ethnically homogeneous population sample with prospective measurements of smoking during pregnancy and childhood maltreatment, and more robust confounding control through modelling of covariates in interaction with both the genetic and environmental factors. Another strength has been the use of diagnostic interview data to measure childhood behavior. Moreover, we also performed adjustments for comorbid ADHD and internalizing problems, which is frequently lacking in both G × E and main effect studies. While we did not find main effects of the genetic variants (which may be due to methodological and/or clinical differences with the original studies), we did observe clear G × E interactions, which points to the importance of this field of study and implies that G × E’s (as part of the broad sense heritability model) might be able to explain part of the so-called ‘missing heritability’ [66, 67]. Of note, ALSPAC is one of the samples used in the GWAS meta-analysis of antisocial behavior by Tielbeek et al. [12]. However, since we failed to replicate the genetic main effect of the female-only SNPs implicated by that study, the meta-analytic genome-wide signals for these SNPs may be driven by the other cohorts in that study. While, on average, antisocial behavior levels were low (as expected in a population cohort), we observed relative effect sizes of moderate-to-large magnitude resulting from common genetic variants and environmental exposures, emphasizing the clinical relevance of these results.

Nevertheless, we need to acknowledge limitations of the present study. First, the use of singular genetic variants does not necessarily provide a comprehensive picture of G × E interactions as the genetic architecture of antisocial behavior is expected to be of a complex nature [6, 12]. Alternative approaches to address this issue include the use of polygenic risk scores, gene-set (for example combining all genetic variants of a specific pathway), or gene-based (i.e., combining all variants related to a gene) analyses rather than singular variants. Nevertheless, we were able to identify different genetic loci that are likely to be of relevance given their implication as GWAS top hits. Furthermore, the top SNPs identified by the Rautiainen et al. GWAS were located only about 8 Mb distance (6p21.2) from the major histocompatibility complex (MHC) region at chromosome 6. The MHC region is highly polymorphic, displays extended LD structures and numerous disease associations have been reported for this region [68]. However, as reported by Rautiainen et al., there was no LD between the identified top SNPs at 6p21.2 and SNPs showing up at the MHC region [13]. Finally, maternal self-report measures of smoking during pregnancy and maltreatment, although measured prospectively may be subject to underreporting due to social desirability bias, which may affect the accuracy of effect estimates.

## Conclusions

We studied sex-stratified G × E interactions in relation to antisocial behavior in a large population cohort and found interactions between recently (GWAS-)implicated variants and well-known environmental adversities. In males, G × E interactions with smoking during pregnancy were observed, which may be related to specific leucine-rich repeat protein networks involved in neurodevelopment. In females, G × E interactions with childhood maltreatment were found for one GWAS top SNP and *MAOA*. We were, however, unable to replicate other previously reported G × E interactions involving the *MAOA* gene. We conclude on a more general level that G × E studies do, indeed, contribute valuable information about the multifactorial nature of antisocial behavior, and we support the notion that well-powered GWASs provide more robust variants for G × E studies than classical candidate genes. Future studies should, in addition to GWAS top hits, incorporate polygenic, multimarker approaches, while addressing statistical robustness and potential sex differences when studying G × E interactions related to antisocial behavior.

## Electronic supplementary material

Below is the link to the electronic supplementary material.

Supplementary material 1 (DOCX 22 KB)
