# Supplementary material for: Interplay between genome-wide implicated genetic variants and environmental factors related to childhood antisocial behavior in the UK ALSPAC cohort
Source: Eur Arch Psychiatry Clin Neurosci. 2018 Dec 19;269(6):741–52. doi: 10.1007/s00406-018-0964-5 (PMC6689282; doi:10.1007/s00406-018-0964-5)
Supplement: Supplementary file 1 — Supplementary material 1 (DOCX 22 KB) [file 406_2018_964_MOESM1_ESM.docx]

**Interplay between genome-wide implicated genetic variants and environmental factors related to childhood antisocial behavior in the UK ALSPAC cohort.**

**Supplementary Material**

Authors:

I. Hyun Ruisch^a^*, M.D.

Andrea Dietrich^a^, Ph.D.

Jeffrey C. Glennon^b^, Ph.D.

Jan K. Buitelaar^b,c^, M.D., Ph.D.

Pieter J. Hoekstra^a^, M.D., Ph.D.

Affiliations:

^a^ University of Groningen, University Medical Center Groningen, Department of child and adolescent psychiatry, Hanzeplein 1, 9713GZ Groningen, The Netherlands.

^b^ Department of Cognitive Neuroscience, Donders Institute for Brain, Cognition and Behaviour, Radboud University Medical Center, Geert Grooteplein Zuid 10, 6525GA Nijmegen, The Netherlands.

^c^ Karakter Child and Adolescent Psychiatry University Centre, Reinier Postlaan 12, 6525 GC Nijmegen, The Netherlands.

Contact information and corresponding author:

** Corresponding author:* I. H. Ruisch, *e-mail address*: [i.h.ruisch@umcg.nl](mailto:i.h.ruisch@umcg.nl), *telephone*: +31 50 361 61 61

*E-mail addresses of co-authors:* [a.dietrich@accare.nl](mailto:a.dietrich@accare.nl) (A. Dietrich), [j.glennon@donders.ru.nl](mailto:j.glennon@donders.ru.nl) (J. C. Glennon), [jan.buitelaar@radboudumc.nl](mailto:jan.buitelaar@radboudumc.nl) (J. K. Buitelaar), [p.hoekstra@accare.nl](mailto:p.hoekstra@accare.nl) (P. J. Hoekstra).

**Keywords:** ALSPAC, antisocial behavior, aggression, gene-environment interaction, maltreatment, smoking during pregnancy.

**Acknowledgements:** We are extremely grateful to all the families who took part in this study, the midwives for their help in recruiting them, and the whole ALSPAC-team, which includes interviewers, computer and laboratory technicians, clerical workers, research scientists, volunteers, managers, receptionists and nurses. The UK Medical Research Council and Wellcome (Grant ref: 102215/2/13/2) and the University of Bristol provide core support for ALSPAC. A comprehensive list of grants funding is available on the ALSPAC-website (<http://www.bristol.ac.uk/alspac/external/documents/grant-acknowledgements.pdf>). Genotype data was generated by Sample Logistics and Genotyping Facilities at Wellcome Sanger Institute and LabCorp (Laboratory Corporation of America) using support from 23andMe. This publication is the work of the authors and this research is supported by the European Community's Seventh Framework Programme (FP7/2007-2013) under grant agreement no. 603016 (MATRICS).

**Table S1: Genotype statistics for included SNPs:**

| SNP | Chromosome: basepair (GRCh37) | MAF | HWE | Call rate | Genotype frequencies (N subjects with complete data) |
| --- | --- | --- | --- | --- | --- |
| rs4714329 | 6: 40273457 | 0.40 | 0.51 | >0.99 | GG: 405  GA: 1250  AA: 920 |
| rs9471290 | 6: 40260515 | 0.36 | 0.22 | >0.99 | AA: 325  AG: 1226  GG: 1024 |
| rs2764450 | 1: 180242092 | 0.06 | >0.99 | >0.99 | TT: 6 (excluded from main analyses)  TC: 280  CC: 2120 |
| rs11215217 | 11: 114689701 | 0.07 | 0.87 | 0.99 | TT: 15 (excluded from main analyses)  TC: 292  CC: 2095 |

SNP: single nucleotide polymorphism. MAF: minor allele frequency. HWE: Hardy-Weinberg equilibrium exact test P-value.

**Table S2: GxE-interactions in males with SNPs coded as additive model:**

| Contrast |  | Males |  |
| --- | --- | --- | --- |
|  | **N** | **IRR (95%-CI)** | **P** |
| rs4714329 GA x smoking during pregnancy  rs4714329 GG x smoking during pregnancy | 2547 | 1.02 (0.72-1.44)  1.96 (1.25-3.08) | 0.93  0.0033 |
| rs4714329 GA x maltreatment  rs4714329 GG x maltreatment | 1431 | 0.88 (0.59-1.32)  0.70 (0.40-1.20) | 0.54  0.20 |
| rs9471290 AG x smoking during pregnancy  rs9471290 AA x smoking during pregnancy | 2547 | 0.98 (0.69-1.40)  2.17 (1.40-3.36) | 0.93  0.0005 |
| rs9471290 AG x maltreatment  rs9471290 AA x maltreatment | 1431 | 0.90 (0.61-1.33)  1.22 (0.65-2.31) | 0.61  0.54 |

GxE: gene by environment interaction. SNP: single nucleotide polymorphism. IRR: incidence rate ratio. All analyses were adjusted for socioeconomic status, single parent status, and the first 10 genetic principal components, including covariate interaction terms for the GxE-models.

**Table S3: Frequencies of childhood antisocial behavior scores (N subjects with complete data):**

| Score | N | % | Score | N | % |
| --- | --- | --- | --- | --- | --- |
|  | **Males (recoded)** | |  | **Females (recoded)** | |
| 0 | 1704 | 66.17% | **0** | 1682 | 69.73% |
| 1 | 510 | 19.81% | **1** | 486 | 20.15% |
| 2 | 186 | 7.22% | **2** | 159 | 6.59% |
| 3 | 87 | 3.38% | **3+** | 85 | 3.52% |
| 4+ | 88 | 3.42% |  |  |  |

**Childhood antisocial behavior score distribution:** our outcome was defined as childhood antisocial behavior, which was measured as mother rated CD symptom scores. These scores consisted of count data with positive skew and overdispersion, as can be observed from the summary statistics in Table 1. To slightly reduce excess variance and avoid low cell counts, we recoded counts N<50 as the score level that originally included the most subjects. This way we recoded 32 males with a score ≥4 as 4, and 35 females with a score ≥3 as 3.

**Table S4: Gene-environment correlations:**

| Polymorphism | rGE with smoking during pregnancy | rGE with maltreatment |
| --- | --- | --- |
| rs4714329 GG (males) | ρ=-0.01, P=0.75 | ρ=-0.02, P=0.38 |
| rs9471290 AA (males) | ρ=0.02, P=0.16 | ρ=0.01, P=0.77 |
| rs2764450 TC (females) | ρ=-0.01, P=0.57 | ρ=-0.01, P=0.67 |
| rs11215217 TC (females) | ρ=-0.02, P=0.14 | ρ<0.01, P=0.85 |
| MAOA-L (males) | ρ>-0.01, P=0.83 | ρ=0.02, P=0.31 |
| MAOA-HL/HH (females) | ρ=0.02, P=0.27 | ρ=-0.02, P=0.42 |

rGE: gene-environment correlation (Spearman’s rho).

**Table S5: Sex- and genotype-stratified environmental main effects in relation to childhood antisocial behavior.**

| Environment | Genotype | N | IRR (95%-CI) | P |
| --- | --- | --- | --- | --- |
| Smoking during pregnancy (males) | rs4714329-GG | 398 | 2.42 (1.65-3.55) | <0.0001 |
|  | rs4714329-GA/AA | 2149 | 1.30 (1.08-1.55) | 0.0046 |
|  | rs9471290-AA | 321 | 2.65 (1.84-3.82) | <0.0001 |
|  | rs9471290-AG/GG | 2226 | 1.25 (1.04-1.49) | 0.0180 |
| Maltreatment (females) | rs11215217-CC | 1125 | 2.24 (1.82-2.76) | <0.0001 |
|  | rs11215217-TC | 160 | 1.07 (0.71-1.61) | 0.76 |
|  | *MAOA-*LL | 138 | 0.87 (0.51-1.48) | 0.61 |
|  | *MAOA-*HL | 520 | 2.52 (1.88-3.38) | <0.0001 |
|  | *MAOA-*HH | 477 | 1.62 (1.19-2.20) | 0.0020 |

IRR: incidence rate ratio. MAOA; monoamine oxidase A. MAOA-L/H: *MAOA* low/high activity allele. ADHD: attention-deficit/hyperactivity disorder. All analyses were adjusted for socioeconomic status, single parent status, and the first 10 genetic principal components, including covariate interaction terms for the GxE-models.
